# PReS-FINAL-2114: The leptin and adiponectin as biomarkers of atherosclerosis in juvenile rheumatic arthritis

**DOI:** 10.1186/1546-0096-11-S2-P126

**Published:** 2013-12-05

**Authors:** V Opoka-Winiarska, J Tabarkiewicz, A Emeryk

**Affiliations:** 1 Dept. of Paediatric Pulmonology and Rheumatology, Medical University of Lublin, Poland; 2 Department of Clinical Immunology, Medical University of Lublin, Poland, Lublin, Poland; 3 Dept. of Paediatric Pulmonology and Rheumatology, Medical University of Lublin, Lublin, Poland

## Introduction

Rheumatic diseases in adults are associated with increased incidence of cardiovascular disease. Compared to many publications related to the risk of atherosclerosis and its complications in patients with rheumatoid arthritis, only few observations have been published on the subject of patients with idiopathic juvenile arthritis (JIA).

## Objectives

The aim of the study was assessing the early vascular disorders associated with atherosclerosis in children with juvenile idiopathic arthritis based on analysis of biomarkers of subclinical atherosclerosis. As biomarkers associated with early atherosclerosis adiponectin and leptin were selected. Because of the possible association between the evaluated biomarkers and inflammation process of joints, the study was conducted in patients during treatment, but in period without disease activity, in order to eliminate the direct impact of arthritis on the test results. Influence of the onset type, duration of the disease and the treatment on evaluated biomarkers and their association with factors connected with cardiovascular risk such as lipid levels and BMI percentile were analyzed.

## Methods

The study was performed in 56 children with JIA, 4-16 years old, without disease activity, during therapy with at least one disease modifying drug, with or without glucocorticoid (at a dose of ≤ 10 mg/day or ≤ 0.2 mg/kg body weight) and 19 healthy children as a control group. 25 patients had oligoarticular onset of disease, 21 - polyarticular onset, 8 had enthesitis-related arthritis and 2 had systemic onset of JIA.

Analysis of blood count, ESR, CRP, cholesterol, triglycerides, LDL and HDL cholesterol and artherosclerosis biomarkers were assessed in all examined children. The concentrations of leptin, adiponectin were made by enzyme immunoassay method using the kits for the quantitative tests in blood serum.

## Results

There were no significant differences between the concentrations in the blood adiponectin, leptin in patients with JIA and control groups. There were no also differences in the parameters between patients with different forms of the disease.

The parameters in the early period JIA (≤ 1 year) and later (> 1 year) were not differenced significant. In the group of children with JIA, a positive correlation between leptin levels and duration of the disease was found. There were no differences in the parameters between the groups of patients treated and not treated with methotrexate, etanercept or glucocorticoids. There were no negative effects of therapy with glucocorticoids and methotrexate on assessed biomarkers. Serum leptin concentrations in the whole study group were significantly higher in girls than in boys. There was a positive correlation between serum leptin and parameters of nutritional status.

## Conclusion

The lack of differences between the biomarkers of early atherosclerosis in group of JIA patients compared to the control group, indicates a reduced risk of cardiovascular diseases, in terms of effective therapy.

The type of treatment do not have impact on the biomarkers, which means that did not increase the risk of vascular disease, the treatment efficacy is more important than the type of used drug. The subtype of the disease also had no effect on biomarkers.

## Disclosure of interest

None declared.

